# Prognostic value of ^18^F-FDG PET/CT-based radiomics combining dosiomics and dose volume histogram for head and neck cancer

**DOI:** 10.1186/s13550-023-00959-6

**Published:** 2023-02-13

**Authors:** Bingzhen Wang, Jinghua Liu, Xiaolei Zhang, Zhongxiao Wang, Zhendong Cao, Lijun Lu, Wenbing Lv, Aihui Wang, Shuyan Li, Xiaotian Wu, Xianling Dong

**Affiliations:** 1 Department of Biomedical Engineering, Chengde Medical University, Chengde, Hebei China; 2 Department of Nursing, Chengde Central Hospital, Chengde, Hebei China; 3 Department of Nursing, Faculty of Medicine and Health Sciences, Universiti Putra Malaysia, Serdang, Malaysia; 4 Department of Radiology, The Affiliated Hospital of Chengde Medical University, Chengde, Hebei China; 5 School of Biomedical Engineering and Guangdong Provincal Key Laboratory of Medical Image Processing, Southern Medical University, Guangzhou, Guangdong China; 6 Department of Electronic Engineering, Information School, Yunnan University, Kunming, Yunnan China; 7 Department of Nuclear Medicine, The Affiliated Hospital of Chengde Medical University, Chengde, Hebei China; 8 Hebei International Research Center of Medical-Engineering, Chengde Medical University, Chengde, Hebei China

**Keywords:** Radiomics, Dosiomics, DVH, Prognosis, PET/CT, Head and neck cancer

## Abstract

**Objectives:**

By comparing the prognostic performance of ^18^F-FDG PET/CT-based radiomics combining dose features [Includes Dosiomics feature and the dose volume histogram (DVH) features] with that of conventional radiomics in head and neck cancer (HNC), multidimensional prognostic models were constructed to investigate the overall survival (OS) in HNC.

**Materials and methods:**

A total of 220 cases from four centres based on the Cancer Imaging Archive public dataset were used in this study, 2260 radiomics features and 1116 dosiomics features and 8 DVH features were extracted for each case, and classified into seven different models of PET, CT, Dose, PET+CT, PET+Dose, CT+Dose and PET+CT+Dose. Features were selected by univariate Cox and Spearman correlation coefficients, and the selected features were brought into the least absolute shrinkage and selection operator (LASSO)-Cox model. A nomogram was constructed to visually analyse the prognostic impact of the incorporated dose features. C-index and Kaplan–Meier curves (log-rank analysis) were used to evaluate and compare these models.

**Results:**

The cases from the four centres were divided into three different training and validation sets according to the hospitals. The PET+CT+Dose model had C-indexes of 0.873 (95% CI 0.812–0.934), 0.759 (95% CI 0.663–0.855) and 0.835 (95% CI 0.745–0.925) in the validation set respectively, outperforming the rest models overall. The PET+CT+Dose model did well in classifying patients into high- and low-risk groups under all three different sets of experiments (*p* < 0.05).

**Conclusion:**

Multidimensional model of radiomics features combining dosiomics features and DVH features showed high prognostic performance for predicting OS in patients with HNC.

**Supplementary Information:**

The online version contains supplementary material available at 10.1186/s13550-023-00959-6.

## Introduction

Head and neck cancer (HNC) was the seventh most common cancer worldwide in 2018, with around 890,000 new cases and 450,000 deaths [1]. However, with advances in treatment technology, the 5-year survival rate for HNC patients is still only 65.9% [2]; local recurrence and distant metastases remain the main causes of treatment failure and death for patients [3–5]. Therefore, it is clinically important to develop a prognostic plan for patients with HNC.

Radiomics is an emerging computer science that extracts high-throughput quantitative features from medical images (e.g. CT, MR, PET, etc.) for clinical analysis through computer technology [6, 7], providing new ideas for mining deeper information in medical images. Radiomics allows the development and refinement of radiomics features that can improve prognostic and predictive models for specific cancers, including HNC [8]. In recent years, there has been an increasing number of radiomics studies on HNC. Xu et al. developed a PET/CT-based subregional radiomics approach to predict progression-free survival in patients with nasopharyngeal carcinoma, demonstrating the prognostic potential of subregional radiomics in nasopharyngeal carcinoma [9]. Keek et al. proposed a multifactorial prognostic model including CT-based radiomic features, TNM8, tumour volume, clinical and biological variables, and demonstrated that the model could predict overall survival (OS) very accurately in patients with advanced head and neck squamous cell carcinoma [10]. Liu et al. found that combining clinicopathological features with pre-treatment PET/CT or post-treatment PET/CT radiomic features substantially improved the prediction of OS and disease-free survival (DFS) in patients with head and neck squamous cell carcinoma [11]. Overall, radiomics remains a promising area of research.

Radiotherapy is still one of the main treatment modalities for HNC [12], and the efficacy of radiation therapy for tumours is closely related to the dose distribution. It has been shown that dose volume histogram (DVH)-related features used to assess radiotherapy planning are effective for predicting progression-free survival in locally advanced nasopharyngeal cancer [13], and for predicting radiation-induced hypothyroidism in patients with nasopharyngeal cancer [14]. However, the DVH features only describe the distribution of dose in general terms during radiotherapy and do not give a specific three-dimensional representation of the radiotherapy dose. The dosiomics method can describe dose distribution by intensity, texture, shape and other dose characteristics with high accuracy, granularity and spatial information, and is an effective method for parameterizing radiotherapy dose distribution [15]. It has been shown that dosiomics features based on dosimetry can be more effective in several directions such as local control after carbon-ion radiotherapy for skull base chordoma [16], prediction of weight loss in the acute phase in lung cancer patients receiving radiotherapy [17] and exploring the interaction between radiation and lymphocytopenia in lung cancer patients [18].

This study combined radiomics, dosiomics and DVH features based on radiotherapy schedule assessment to construct a multidimensional prognostic model for HNC. With this multidimensional prognostic model, this study will investigate whether the inclusion of dose features can enhance the prognostic performance of the Radiomics model.

## Materials and methods

### Patients

**Fig. 1:**
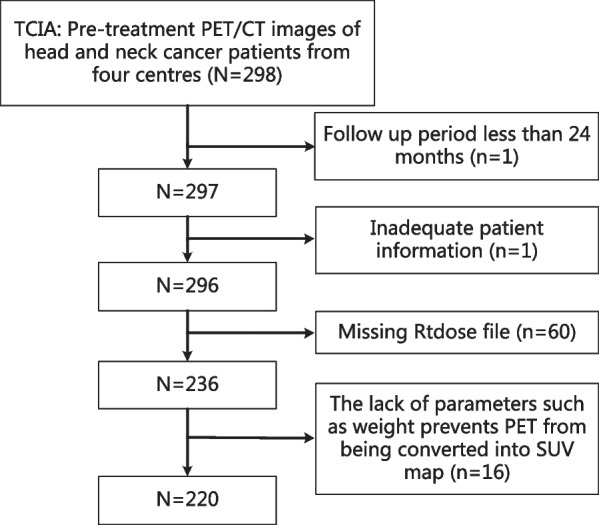
Case selection flow chart

The dataset used in this study was from The Cancer Imaging Archive (TCIA) (http://www.cancerimagingarchive.net) and the original dataset contains ^18^F-FDG PET/CT imaging data from 298 patients at four different institutions in Québec, Canada, outcome data and radiotherapy profiles (RTstruct). The case selection process is shown in Fig. 1, a total of 220 cases were included. Of these, 64 cases were from the Centre hospitalier de l'Université de Montréal (CHUM, labelled CEN1), 75 cases from the Centre hospitalier universitaire de Sherbrooke (CHUS, labelled CEN2), and 55 cases from the Hôpital général juif de Montréal (HGJ, labelled CEN3), and 26 from the Hôpital Maisonneuve Rosemont de Montréal (HMR, labelled CEN4). The characteristics of the 220 patients are shown in Table 1.

**Table 1:** Patients’ characteristics of four centres

Characteristic	CHUM	CHUS	HGJ	HMR	*p*
	64	75	55	26	
Sex^a^					0.715
Male	48 (75%)	55 (73.3%)	45 (81.8%)	20 (76.9%)	
Female	16 (25%)	20 (26.7%)	10 (18.2%)	6 (23.1%)	
Age^b^ (mean (SD))	63.41 (9.26)	64.04 (10.78)	62.53 (10.03)	66.46 (9.01)	0.411
Primary Site ^c^					< 0.001
Nasopharynx	1 (1.6%)	5 (6.7%)	6 (10.9%)	6 (23.1%)	
Oropharynx	57 (89.1%)	56 (74.7%)	36 (65.5%)	11 (42.3%)	
Hypopharynx	1 (1.6%)	1 (1.3%)	3 (5.5%)	6 (23.1%)	
Larynx	0 (0.0%)	13 (17.3%)	8 (14.5%)	3 (11.5%)	
Unknown	5 (7.8%)	0 (0.0%)	2 (3.6%)	0 (0.0%)	
T-stage^c^					0.016
Tx	5 (7.8%)	0 (0.0%)	2 (3.6%)	1 (3.8%)	
T1	8 (12.5%)	6 (8.0%)	11 (20.0%)	2 (7.7%)	
T2	28 (43.8%)	32 (42.7%)	14 (25.5%)	9 (34.6%)	
T3	8 (28.1%)	23 (30.7%)	23 (41.8%)	6 (23.1%)	
T4	5 (7.8%)	14 (18.7%)	5 (9.1%)	8 (30.8%)	
N-stage^c^					0.001
N0	4 (6.2%)	24 (32.0%)	11 (20.0%)	3 (11.5%)	
N1	8 (12.5%)	7 (9.3%)	12 (21.8%)	3 (11.5%)	
N2	45 (70.3%)	41 (54.7%)	31 (56.4%)	15 (57.7%)	
N3	7 (10.9%)	3 (4.0%)	1 (1.8%)	5 (19.2%)	
Stage^c^					0.002
Stage I	0 (0.0%)	1 (1.3%)	1 (1.8%)	0 (0.0%)	
Stage II	2 (3.1%)	11 (14.7%)	3 (5.5%)	3 (11.5%)	
Stage III	7 (10.9%)	13 (17.3%)	21 (38.2%)	2 (7.7%)	
Stage IV	53 (82.8%)	50 (66.7%)	30 (54.5%)	21 (80.8%)	
Unknown	2 (3.1%)	0 (0.0%)	0 (0.0%)	0 (0.0%)	
Therapy^c^					< 0.001
RT	4 (6.2%)	21(28.0%)	3(5.5%)	5(19.2%)	
CRT	60 (93.8%)	54 (72.0%)	52 (94.5%)	21 (80.8%)	
Death^a^					< 0.001
Yes	4(6.2%)	12(16.0%)	6(10.9%)	15(57.7%)	
No	60 (93.8%)	63 (84.0%)	49 (89.1%)	11 (42.3%)	

*RT* Radiotherapy; *CRT* Chemoradiotherapy; *SD* Standard deviation

a: Pearson Chi-square test; b: Kruskal–Wallis test; c: Fisher’s exact test

**Fig. 2:**
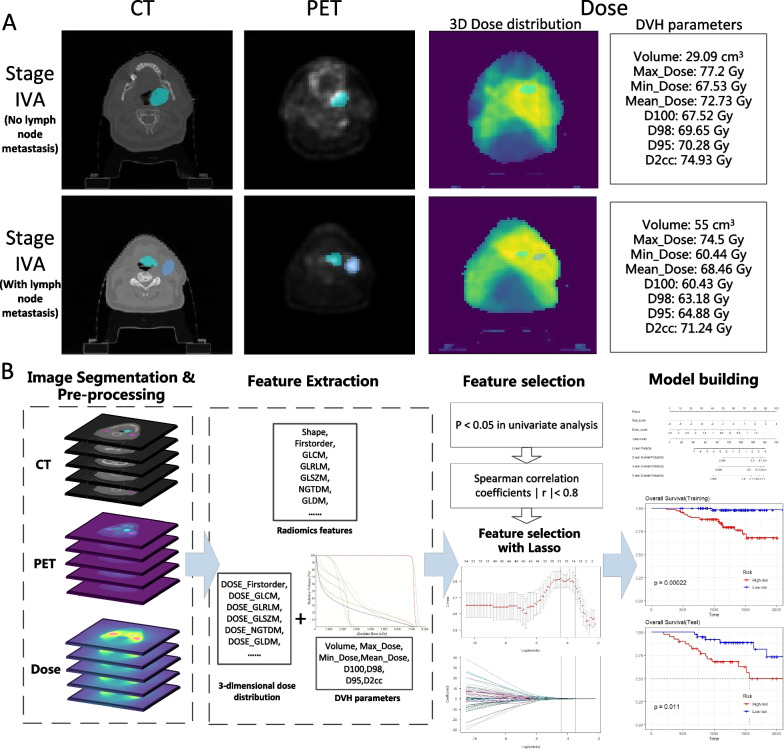
**A** Differences in dose and imaging in patients with the same stage IVA, **B** Overall experimental design of this study (including image segmentation & pre-processing, feature extraction, feature selection and model building)

All patients had pathologically confirmed HNC and underwent ^18^F-FDG PET/CT scans prior to treatment. Of the 220 patients, 37 patients were treated with radiotherapy alone and 183 patients were treated with chemoradiotherapy. PET images had varying pixel sizes of 3.52–5.47 mm, slice thicknesses of 3.27–4 mm, slice spacings of 3.27–4 mm with matrix sizes of 128 or 144, whereas CT images have varying pixel sizes of 0.68–1.37 mm, slice thicknesses of 1.5–3.75 mm, slice spacings of 1.5–3.27 mm and matrix size of 512. As seen in Fig. 2A, there are differences in tumour size, lymph node metastasis and dose distribution in cases with the same Stage IVA. The radiotherapy contours defining the gross tumour volume (GTV) and lymph nodes were drawn by an expert radiation oncologist on a different CT scan dedicated to treatment planning. Similarly, the contours of the RTdose file are based on the CT contours. More details about the patient in Additional file 1.

### Study design

Figure 2B shows the overall experimental design. To test the performance of the model under different training and validation sets, three different training and validation sets, CEN 12 versus 34 (Centre 1 and Centre 2 are used as training sets, Centre 3 and Centre 4 are used as validation sets, labelled CEN 12 vs. 34), CEN 13 versus 24 and CEN 23 versus 14 were used to predict the OS of the cases. The dosiomics feature and the DVH features are collectively referred to as the dose feature in this article. After feature filtering, seven models were constructed for each group: PET, CT, PET+CT, Dose, PET+Dose, CT+Dose, PET+CT+Dose. A nomogram was drawn to provide insight into the relationship between dosiomics features, radiomics features and OS.

### Image pre-processing

Prior to feature extraction, ^18^F-FDG PET images were converted to standard uptake value (SUV) maps [19], and PET, CT, dose matrix and segmentation mask were isotropically resampled using NearestNeighbor interpolation with a resampling voxel size of 1 × 1 × 1 mm^3^.

In this study, the tumour volume was defined as the primary tumour site and the surrounding metastatic lymph nodes (GTV primary+GTV lymph nodes). In addition to the original images, wavelet filtered images of PET, CT, RTdose files and LoG filtered images were included. Wavelet filtered images were obtained by applying a “Coiflet” wavelet transform to the original images using low-pass and high-pass filters in the x, y and z directions, and LoG filtered images by applying a Laplacian of Gaussian filter with different parameters (*σ* = 1, 3, 5).

### Feature extraction

Feature extraction was based on the open-source toolkit PyRadiomics [20] (https://pyradiomics.readthedocs.io/en/latest/). A total of 3384 features were extracted for each case, including 14 shape features extracted from each of the PET and CT images, and 1116 first order features and texture features extracted from the PET, CT, RTdose and 11 filtered images (including 12 × 18 first order features, 12 × 24 Gy level co-occurrence matrix (GLCM) features [21], 12 × 14 Gy level dependence matrix (GLDM) features [22], 12 × 16 Gy level size zone matrix (GLSZM) features [23], 12 × 16 grey level run length matrix (GLRLM) features [24] and 12 × 5 neighbouring grey tone difference Matrix (NGTDM) features [25]). In addition, eight DVH features (Volume, Max_Dose, Min_Dose, Mean_Dose, D100, D98, D95 and D2cc) were extracted based on the Python package dicompyler-core, from the RTstruct file and RTdose file for each case. Thus, 2260 ^18^F-FDG PET/CT-based radiomics features and 1124 dose features were extracted for each case.

### Feature selection

**Fig. 3:**
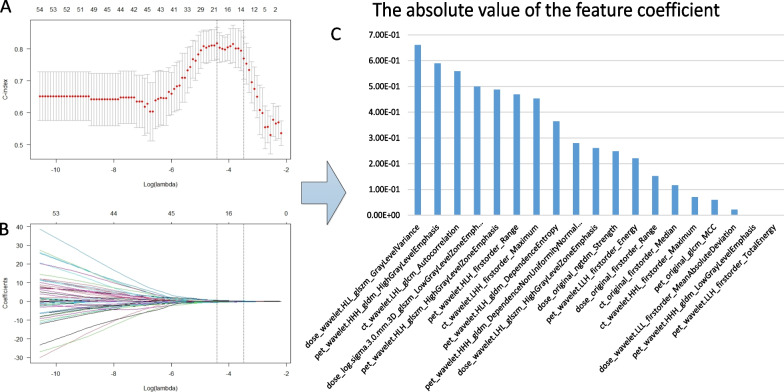
Feature filtering by LASSO when the dataset was divided into CEN12 versus 34. **A** Tuning parameter (λ) selection in the LASSO model used tenfold cross-validation via minimum criteria. **B** LASSO coefficient profiles, A coefficient profile plot was produced against the log (λ) sequence. **C** Weight map of the final incorporated features, values were the absolute values of the feature coefficients, eight features with the highest absolute values of the coefficients were included for further study

The features of each case were Z-score standardized so that the values of each feature were in the same order of magnitude, which was conducive to improving the accuracy of the prognostic model [26]. The PET features, CT features and dose features were screened separately. The included features were first subjected to univariate Cox analysis and those with *p* > 0.05 were removed, and features with a Spearman correlation coefficient greater than 0.8 were considered redundant. The PET,CT,Dose feature sets are then further combined into PET, CT, PET+CT, Dose, PET+Dose, CT+Dose, PET+CT+Dose feature sets, and finally further screened using LASSO and tenfold cross-validation. If the final number of features included was greater than 8, the eight features with the highest absolute value of the feature coefficients were included in the further analysis in order to prevent overfitting of the model. Figure 3 shows the process of filtering features based on LASSO.

### Radiomics signature and dose signature building

A multivariate Cox proportional hazards regression model was constructed to correlate the characteristics and prognosis of OS. Among the features finally included in the PET+CT+Dose model, the radiomics score (Rad-score) and the dose score (Dose-score) were calculated separately for each patient by weighting the selected features by their respective coefficients in a linear combination [27]. The Rad-score includes PET radiomics features and CT radiomics features, and the Dose-score includes dosiomics features and DVH features.

### Identification of high- and low-risk groups of patients

The PET, CT and PET+CT models used the median of the Rad-score as the cut-off value. The Dose model used the median of the Dose-score as the cut-off value, and PET+Dose, CT+Dose and PET+CT+Dose use the median of the sum of the Rad-score and Dose-score as the cut-off value.

### Statistical analysis

All statistical analyses were performed using R software (version 4.2.1). The C-index was used to assess the prognostic performance of the model. KM curves and log-rank tests were then used to assess whether there was a significant difference in survival between the two groups [28]. If the difference in survival analysis was significant (*p* < 0.05), the model was performing well and can successfully separate high- and low-risk groups [29]. A two-sided *p* value < 0.05 was used as the criterion.

The “glment” package was used to complete the LASSO regressions, the “survival” package to complete the univariate Cox and multivariate Cox regressions for the survival analysis, the “corrplot” package to calculate correlation coefficients between features, the “rms” package to plot nomograms and to plot calibration curves, the “survminer” package to plot KM survival curves, and the “compare” package was used to complete the statistical test for the difference between the two C-indexes.

## Results

The results are shown in Table 2. When the training and validation sets of PET+CT+Dose model were divided into CEN 12 versus 34, the C-index of the training set was 0.875 (95% CI 0.802–0.947) and the C-index of the validation set was 0.873 (95% CI 0.812–0.934). When the training and validation sets were divided into CEN 23 versus 14, the C-index for the training set was 0.817 (95% CI 0.725–0.909) and the C-index for the validation set was 0.835 (95% CI 0.745–0.925), which outperformed the rest models. When the training and validation sets were divided into CEN 13 versus 24, the C-index of the model in the training set was 0.819 (95% CI 0.723–0.915), which was also higher than that of the rest models.

**Table 2:** Results for each group of studies

Model	12 versus 34		13 versus 24		23 versus 14	
	Training	Validation	Training	Validation	Training	Validation
*PET*						
C-index	0.811	0.781	0.811	0.761	0.802	0.79
Standard error	0.045	0.054	0.053	0.048	0.05	0.066
*CT*						
C-index	0.811	0.787	0.634	0.668	0.737	0.71
Standard error	0.053	0.06	0.094	0.059	0.06	0.066
*PET+CT*						
C-index	0.859	0.846	0.814	0.762	0.795	0.815
Standard error	0.044	0.041	0.049	0.048	0.044	0.068
*Dose*						
C-index	0.787	0.749	0.722	0.685	0.682	0.623
Standard error	0.049	0.06	0.098	0.055	0.07	0.066
*PET+dose*						
C-index	0.868	0.824	0.817	0.756	0.808	0.777
Standard error	0.028	0.045	0.049	0.05	0.038	0.052
*CT+Dose*						
C-index	0.818	0.807	0.671	0.643	0.768	0.804
Standard error	0.051	0.04	0.099	0.064	0.064	0.051
*PET+CT+Dose*						
C-index	0.875	0.873	0.819	0.759	0.817	0.835
Standard error	0.037	0.031	0.049	0.049	0.047	0.046

### Radiomics signature and dose signature building and validation

**Fig. 4:**
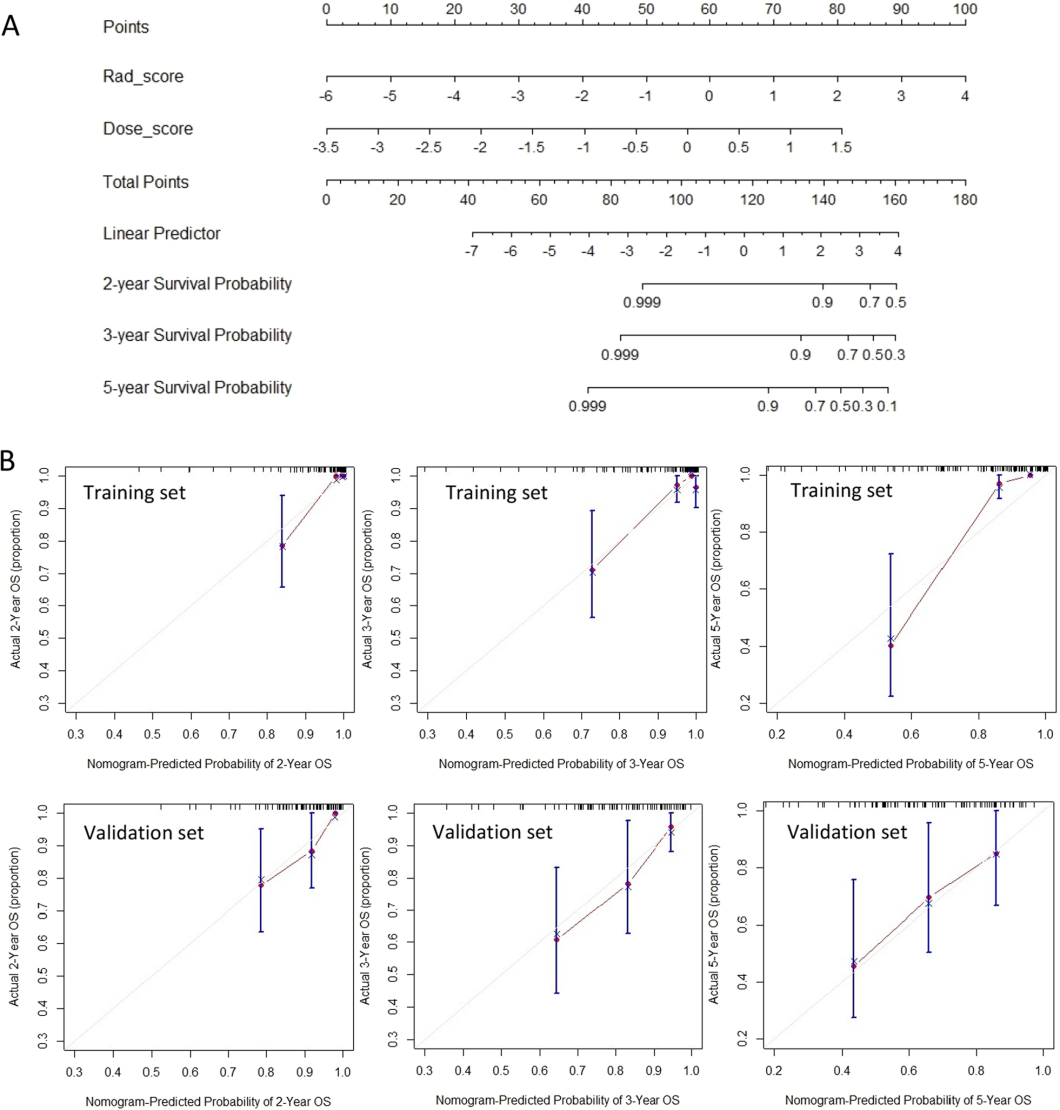
Nomogram and calibration curves. **A** Nomogram of OS when the dataset was divided into CEN12 versus 34. **B** Calibration curves for 2-, 3- and 5-year survival in the training and validation sets

Table 3 shows the final features and feature coefficients included in each model. In the CEN12 versus 34 model, a total of 8 features were included, including 6 PET+CT radiomics features, and 2 dose features. Rad-score and Dose-score were calculated for each patient. Figure 4A shows the nomogram predicting 2-, 3- and 5-year survival based on the CEN 12 versus 34 model, while Fig. 4B shows the calibration curve of the model, where the 45° grey line was the ideal prediction the red line was the nomogram prediction of the model, the more the two overlap, the better the calibration curve was [30]. The final nomogram calibration curves show a high degree of agreement between the predicted and actual results when predicting the 2-year, 3-year and 5-year OS.

**Table 3:** Selected features and their coefficients

Feature name	Coef.
**CEN12 versus CEN34**	
pet_wavelet.HLH_firstorder_Range	0.4694466
pet_wavelet.HLH_gldm_DependenceEntropy	0.3643506
pet_wavelet.HLH_glszm_HighGrayLevelZoneEmphasis	− 0.4874741
pet_wavelet.HHH_gldm_HighGrayLevelEmphasis	− 0.5893292
ct_wavelet.LHL_glcm_Autocorrelation	0.5588674
ct_wavelet.LHH_firstorder_Maximum	0.4527788
dose_log.sigma.3.0.mm.3D_glszm_LowGrayLevelZoneEmphasis	− 0.499172
dose_wavelet.HLL_glszm_GrayLevelVariance	0.6611721
**CEN13 versus CEN24**	
pet_wavelet.LLH_firstorder_Maximum	0.34978373
pet_wavelet.LLH_firstorder_Range	0.10814759
pet_wavelet.HHH_firstorder_Minimum	− 0.398904
ct_original_shape_MinorAxisLength	0.04598108
dose_log.sigma.3.0.mm.3D_glrlm_RunLengthNonUniformity	0.3464844
**CEN23 versus CEN14**	
pet_wavelet.LHH_firstorder_Energy	0.09615666
pet_wavelet.HLL_gldm_SmallDependenceLowGrayLevelEmphasis	− 0.11453625
pet_wavelet.HLH_firstorder_Energy	0.32096706
pet_wavelet.HHH_gldm_DependenceEntropy	0.11999183
ct_wavelet.LLL_glszm_SizeZoneNonUniformityNormalized	− 0.13433928
dose_original_gldm_LargeDependenceHighGrayLevelEmphasis	0.12489405
dose_original_ngtdm_Strength	0.15890523
dose_wavelet.LHL_glszm_GrayLevelVariance	− 0.15117204

### Prognostic value of dose features

**Fig. 5:**
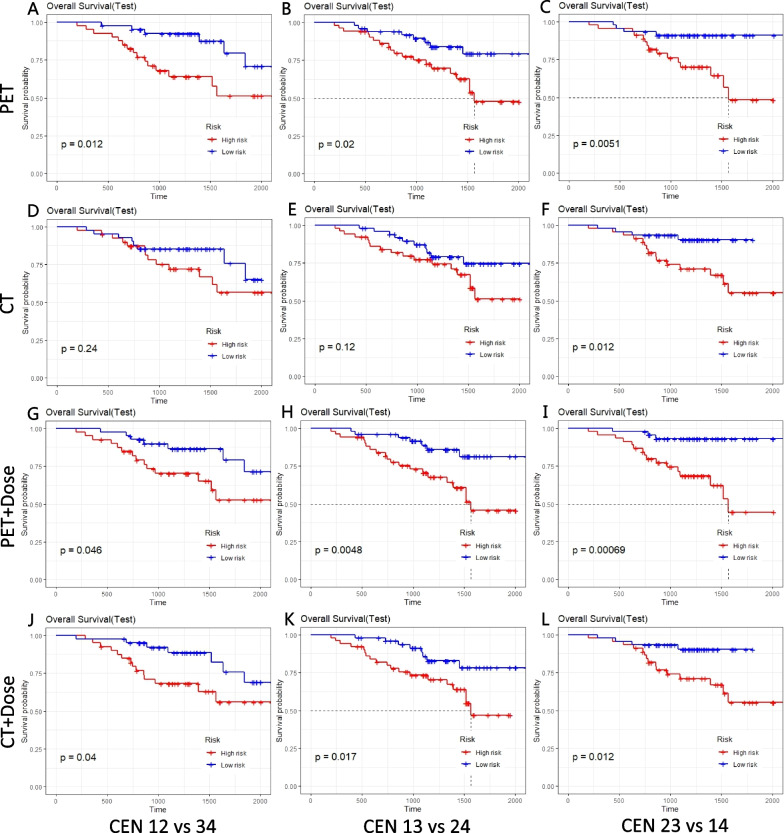
Kaplan–Meier survival analysis using PET,CT,Dose alone

Figure 5 shows in detail the Kaplan–Meier survival curves for each model for different experiments. The Kaplan–Meier survival curves showed that the ^18^F-FDG PET-based radiomics feature (Fig. 5A–C) was significantly different in the classification of high- and low-risk groups (*p* < 0.05), while the CT-based radiomics feature was not significantly different in the classification of high- and low-risk groups in the CEN 12 versus 34 group (*p* = 0.24) (Fig. 5D), nor in the CEN 13 versus 24 group (*p* = 0.12) (Fig. 5E), failure to classify patients into high- and low-risk groups. Thus it can be seen that prognosis was not good when CT features alone were used.

Figure 5G–L shows the Kaplan–Meier survival curves for the PET+Dose model and CT+Dose model in three different dataset partitioning experiments. There was a significant difference in the classification of high- and low-risk groups between the two models (*p* < 0.05). Compared to Fig. 5A–F, it can be seen that incorporating the dose features could improve the prognostic performance of the model.

### Comparison of OS prognostic models

**Fig. 6:**
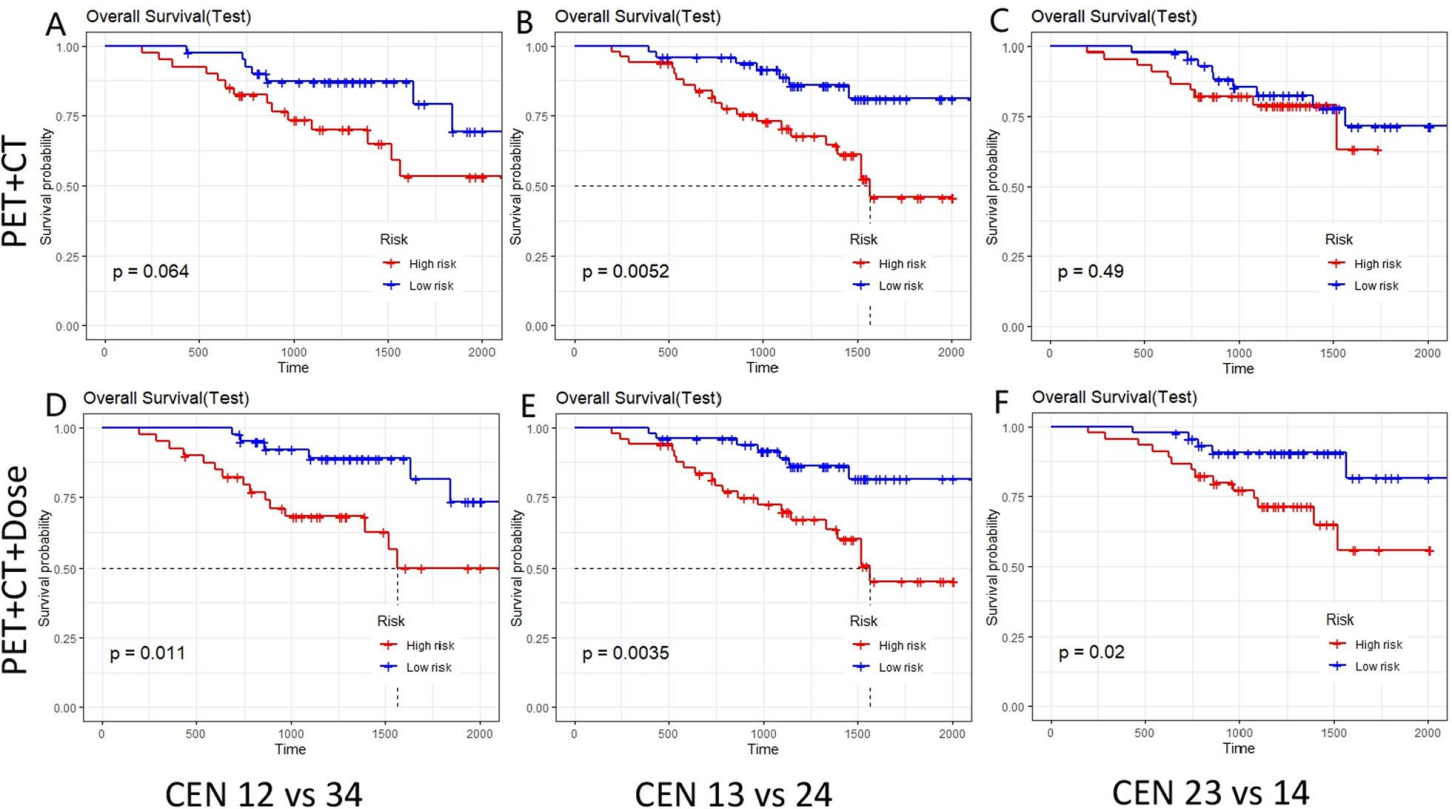
Kaplan–Meier survival analysis of PET+CT versus PET+CT+Dose

In Fig. 6, the KM survival curves for the PET+CT model as well as the PET+CT+Dose model were shown for three different datasets. It was apparent that the PET+CT model did not show a significant difference in the classification of high- and low-risk groups in the CEN 23 versus 14 group (Fig. 6C *p* = 0.49), whereas the PET+CT+Dose model showed a significant difference in the classification of high- and low-risk groups in each group (Fig. 6D–F *p* < 0.05). This suggests that the PET+CT+Dose model was able to significantly improve prognostic performance compared to the conventional PET+CT model. The results from Table 2 also show that the PET+CT model has a C-index of 0.846 (95% CI 0.766–0.926), 0.762 (95% CI 0.668–0.856) and 0.815 (95% CI 0.682–0.948) for the validation set in three different dataset divisions, respectively, which was overall inferior to the PET+CT+Dose model.

## Discussion

Radiotherapy is one of the main treatment modalities for HNC [31]. In this study, a multidimensional prognostic model for HNC was constructed by incorporating PET- and CT-based radiomics features, as well as DVH features and dosiomics features. The results show that the multidimensional prognostic model is superior to that of the conventional radiomics-based prognostic model. This study can illustrate that dosiomics-based features can improve the prognostic performance for predicting OS in patients with HNC.

In this study, although the dose features alone did not perform well prognostically compared to traditional radiomics, the multidimensional model performed better prognostically, suggesting that the radiomics features and the dose features are somewhat complementary. Among the conventional radiomics-based prognostic models for HNC, PET-based radiomics features showed better prognostic performance compared to CT. Also, PET or CT models incorporating dose features outperformed models using only PET or CT images, suggesting that dose features can enhance the prognostic performance of HNC. In the validation set of CEN 12 versus 34 group, the prognostic performance of the models incorporating dose features was not better than that of the models not incorporating dose features. This due to the fact that a total of five features were only included in this group, including 4 radiomics features and 1 dose feature, a lower number of features included than the other models. This should account for the poor performance in this group. However, as the difference is not significant, we do not consider this result sufficient to overturn the conclusion.

The focus of this study was to explore the impact of the inclusion of dosiomics features and DVH features on the prognosis of HNC. In contrast to other studies, this study transformed the RTdose files by filtering them as well. Therefore, more features were extracted from each case, and the number of features extracted was significantly higher than other similar studies, and the more detailed the features extracted, the more useful it was to characterize the tumour information [32]. In this study, a step of univariate Cox analysis followed by Spearman correlation coefficient analysis was used. After the final elimination of the multicollinearity features by LASSO, only the eight features with the largest absolute values of the feature coefficients were selected in order to prevent overfitting due to the inclusion of too many features. As can be seen in Table 3, one or more dose features were included in the multidimensional prognostic model. Meanwhile, the selected features were from the filtered images, which is similar to the results obtained in some other studies [7, 10, 33–36], indicating that the filtered images are able to reveal information that is difficult to convey in the original images.

Table 4 shows the results compared with other studies. Wu et al. incorporated dosiomics features, and the first step of feature selection in that study, in a univariate Cox of features, removed every feature with a C-index greater than 0.61, leaving only 62 features after screening the 1793 features extracted from each case, and the subsequent features were analysed using principal component analysis [37]. While this removed a large number of features, we found in a follow-up study that this approach also removed some important features. And the final results of this study filtered out the extracted clinical features, resulting in a final inclusion of clinical parameters that were inferior to those that were not included. The study by Lv et al. used a multimodal multi-level fusion radiomics protocol for the prognosis of HNC, and although the final results were not as good as other studies, the final inclusion of features was also significantly less than other studies [38]. Vallières et al. used a combination of logistic regression and random forest [39], and Wang et al. constructed a multi-classifier, multi-objective and multimodality model for the prognosis of HNC [40]. These differences in methodology can all lead to differences in the results of the study.

**Table 4:** Comparison of the results of this study with other studies

	Our study	Wenbing Lv	Martin Vallières	Aiqian Wu	Kai Wang
C-index	0.759–0.873 (OS)	0.7 (OS)	0.76 (OS)	0.66 (LR)	0.76 (AUC)
Modality	PET, CT, RT dose	PET, CT	PET, CT	PET, CT, RT dose	PET, CT
Voxel size	1 mm^3^	1 mm^3^	1, 2, 3, 4, 5 mm^3^	3 mm^3^	1, 2, 3, 4 mm^3^
Number of features extracted	3384	254	3230	1793	257
Number of features included	8	< 5	4–7	8	Not report

The strength of this study is that the features ultimately included in this study were significantly associated with prognosis in terms of OS compared to other studies. The reasons for this are manifold; on the one hand, we extracted the most detailed filtered images possible for each case, which included filtered images of RTdose, so that 3384 features were extracted for each case, a significantly higher number of features than in other studies [41]. On the other hand, we did not cut features heavily by increasing the feature screening bounds at a particular step when performing univariate Cox analysis and Spearman's correlation coefficient screening of features, which allowed important features to be retained [42, 43], and ultimately the eight features with the largest absolute values of coefficients were selected when LASSO was used to eliminate multicollinearity features. In addition to the reasons mentioned above, the dose features included in this study, although not included in the clinical features, also enhance prognostic accuracy very well, and all these reasons make the results of this study higher than other studies.

This study has some limitations. First, because the PET, CT and RTdose files all extracted different filtered images, 3384 features were ultimately incorporated for each case, and of these, only eight DVH features [44], so the present model does not demonstrate whether DVH features improve prognostic accuracy. Due to platform limitations, we were not able to extract the conformity index, homogeneity index, V20, V30, V40, V50 and V60 parameters, which also limits the prognostic power of the DVH features. Secondly, although this model is multicentre data, there are only 220 cases, and more cases are needed to improve the prognostic accuracy [45]. Therefore, in future studies, we will try to increase the number of cases as much as possible. In addition, three different training and validation sets were divided in this study. Among the features included in these three different training sets, there were no identical features, and therefore, it was not possible to determine which feature had better prognostic value for HNC. This is related to the fact that we extracted a large number of features in each case. Because of the large number of features, more features capable of presenting tumour information were included, but when the number of features included was greater than eight, we only selected the eight features with the largest absolute value of the feature coefficients, which also led to the inclusion of different features in the three different sets of experiments. This is something that we need to keep exploring in our future research.

## Conclusion

In summary, multidimensional prognostic model combining the radiomics features, DVH features and dosiomics features can be used for prognostic prediction of multicentre HNC, and the prognosis is superior to that of the ^18^F-FDG PET/CT-based radiomics model. Therefore, the importance of dosiomics features and DVH features in the survival prediction of tumours cannot be ignored.

## Supplementary Information

**Additional file 1: Fig. S1.** Principal component analysis of patients from different centres. **Patient datasets.** Detailed data from four centre cases.

## Data Availability

The data presented in this study are available on request from the corresponding author.

## Abbreviations

DVH: Dose volume histogram
HNC: Head and neck cancer
OS: Overall survival
PET: Positron emission computed tomography
CT: Computed tomography
MR: Magnetic resonance imaging
LASSO: Least absolute shrinkage and selection operator
KM: Kaplan–Meier
GLCM: Grey level co-occurrence matrix
GLDM: Grey level dependence matrix
GLSZM: Grey level size zone matrix
GLRLM: Grey level run length matrix
NGTDM: Neighbouring grey tone difference Matrix
