# Supplementary material for: Prognostic value of 18F-FDG PET/CT-based radiomics combining dosiomics and dose volume histogram for head and neck cancer
Source: EJNMMI Res. 2023 Feb 13;13:14. doi: 10.1186/s13550-023-00959-6 (PMC9925656; doi:10.1186/s13550-023-00959-6)
Supplement: Supplementary file 1 — Additional file 1: Fig. S1. Principal component analysis of patients from different centres. Patient datasets. Detailed data from four centre cases. [file 13550_2023_959_MOESM1_ESM.docx]

# Batch effect detection

A total of 220 cases from four centres were included in this study, with the first centre containing 64 cases, the second 75 cases, the third 55 cases and the fourth 26 cases. Prior to data analysis, a principal component analysis was performed on the characteristics, from which a batch effect was detected for the different centre features. The final results were shown in Fig.1, from which it can be seen that the batch effect for the four centres was small and within acceptable limits.


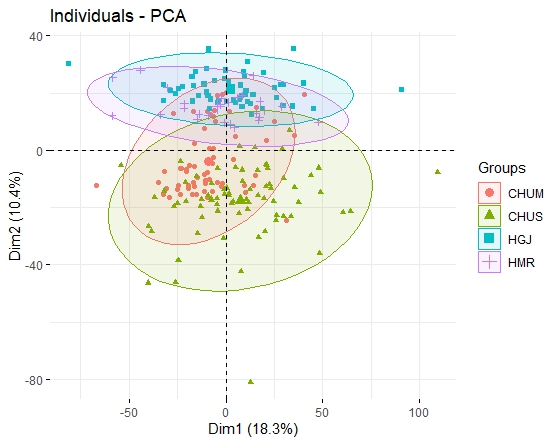


**Fig.S1**. Principal component analysis of patients from different centres

As the focus of this study is on whether the inclusion of dose features has an effect on the prognosis of head and neck cancer, we divided the experimental data into three different training and test sets in the subsequent experiments, namely CEN 12 vs 34 (Centre 1 and Centre 2 are used as training sets, Centre 3 and Centre 4 are used as validation sets, labelled CEN 12 vs 34), CEN 13 vs 24 and CEN 23 vs 14.Also, given that the batch effect of multicentre data can only be weakened and not eliminated, we did not use the COMBAT method to remove the batch effect here.

# Patient datasets

## Centre hospitalier de l'Université de Montréal

**Patient Population**

This cohort is composed of 64 patients with primary squamous cell carcinoma of the head-and-neck (stage II-IVb) treated between 2009 and 2013 at Centre hospitalier de l'Université de Montréal, Montreal,QC. Included patients were treated with curative intent with radiation alone or with chemo-radiation. Patients with recurrent head-and-neck cancer or with metastases at presentation, and patients receiving palliative treatment were excluded from the study. The median follow-up period of the cohort was 40 months (range:11-66). Patients that did not develop a locoregional recurrence or distant metastases during the follow-up period and that had a follow-up time smaller than 24 months were also excluded from the study. The study has been approved by the institutional review board of Centre hospitalier de l'Université de Montréal.

**Treatment Details**

Most patients (94 %) underwent concurrent platinum based chemotherapy and radiotherapy. All patients received an IMRT type radiation (sliding window IMRT or tomotherapy) consisting of 70 Gy of radiation in 33 fractions. Immobilisation device included a thermoplastic mask of the head and shoulder fixed to the treatment table.

**^18^F-FDG PET/CT Scans**

All 64 eligible patients had FDG-PET and CT scans done on a hybrid PET/CT scanner (Discovery STE, GE Healthcare) within 66 days before treatment (median: 12 days). For the PET portion of the FDG-PET/CT scan, a median of 315 MBq (range: 199-3182) was injected intravenously. Imaging acquisition of the head and neck was performed using multiple bed positions with a median of 300 s (range: 120-420) per bed position. Attenuation corrected images were reconstructed using an ordered subset expectation maximization (OSEM) iterative algorithm and a medianspan (axial mash) of 3 (range:3-5). The median FDG-PET slice thickness resolution was 4 mm (range: 3.27-4) and the median in-plane resolution was 4×4 mm^2^ (range: 3.52-5.47). For the CT portion of the FDG-PET/CT scan, a median energy of 120 kVp (range: 120-140) with a median exposure of 350 mAs (range:5-350) was used. The median CT slice thickness resolution was 1.5 mm (range:1.5-3.75) and the median in-plane resolution was 0.98×0.98 mm^2^ (range: 0.98-1.37). All patients received their FDG-PET/CT scan dedicated to the head and neck area right before their planning CT scan, in the same position with the immobilisation device. Contours defining the gross tumour volume (GTV) and lymph nodes were drawn by an expert radiation oncologist on the planning CT scan. The contours were then propagated to the FDG-PET/CT scan reference frame using deformable registration with the software MIM® (MIM software Inc., Cleveland, OH) to ensure proper coverage.

## Centre hospitalier universitaire de Sherbrooke

**Patient Population**

This cohort is composed of 75 patients with primary squamous cell carcinoma of the head-and-neck (stage I-IVb) treated between 2007 and 2014 at Centre hospitalier universitaire de Sherbooke, Sherbrooke, QC. Included patients were treated with curative intent with radiation alone or with chemo-radiation. Patients with recurrent head-and-neck cancer or with metastases at presentation, and patients receiving palliative treatment were excluded from the study. The median follow-up period of the cohort was 44 months (range: 8-93). Patients that did not develop a locoregional recurrence or distant metastases during the follow-up period and that had a follow-up time smaller than 24 months were also excluded from the study. The study has been approved by the institutional review board of Centre hospitalier universitaire de Sherbooke.

**Treatment Details**

All patients have had a pathological confirmation of squamous cell carcinoma and imaging examination for tumor staging before all treatments. All those patients have had a treatment position PET imaging. The PET images have been merged with dosimetry CT imaging, and the dosimetry plan has been performed with teraplan for 3D-conformal technique and pinnacle system for IMRT. The 3D-conformal technique has been used for all patients before 2008, and since 2008, all patients have been treated by IMRT. The treatment approaches consisted of either radiotherapy alone or radiotherapy with concurrent chemotherapy or concurrent Cetuximab. The treatment dose varied according to the tumor staging. The patients with T1 glottic laryngeal cancer have been treated mostly by 2.5 Gy daily for total dose of 50Gy, some patients have been treated with daily dose of 2.25 Gy for 63 Gy totally. All other patients with T1, T2, N0 cancers have been treated with standard fractionated radiation schedules of 60-66 Gy; for the patients with T3-4, or N+, the treatment dose varied from 68.8 Gy in 32 fractions to 70 Gy in 33 fractions. All treatments have been performed by 6 MV linear accelerator. The concurrent chemotherapy was either cisplatin 100 mg/m^2^ at D1, D22 & D43, or cisplatin 40 mg/m^2^, weekly. According to the consideration of the oncologist, some patients have been treated by radiotherapy associated with Cetuximab, due to the problems of kidney function, audition, elder or weak general performance status.

**^18^F-FDG PET/CT Scans**

All 102 eligible patients had FDG-PET and CT scans done on a hybrid PET/CT scanner (GeminiGXL 16, Philips) within 54 days before treatment (median: 19 days). For the PET portion of the FDG-PET/CT scan, a median of 325 MBq (range: 165-517) was injected intravenously. Imaging acquisition of the head and neck was performed using multiple bed positions with a median of 150 s (range: 120-151) per bed position. Attenuation corrected images were reconstructed using a LOR-RAMLA iterative algorithm. The FDG-PET slice thickness resolution was 4 mm and the median in-plane resolution was 4×4 mm^2^ for all patients. For the CT portion of the FDG-PET/CT scan, a median energy of 140 kVp (range: 12-140) with a median exposure of 210 mAs (range: 43-250) was used. The median CT slice thickness resolution was 3 mm (range: 2-5) and the median in-plane resolution was 1.17×1.17 mm^2^ (range: 0.68-1.17). Contours defining the gross tumour volume (GTV) and lymph nodes were drawn by an expert radiation oncologist in a radiotherapy treatment planning system. For 64 of the 75 patients, the radiotherapy contours were directly drawn on the CT scan of the FDG-PET/CT scan. For 11 of the 75 patients, the radiotherapy contours were drawn on a different CT scan dedicated to treatment planning. In the latter case, the contours were propagated to the FDG-PET/CT scan reference frame using deformable registration with the software MIM® (MIM software Inc., Cleveland, OH).

## Hôpital général juif de Montréal

**Patient Population**

This cohort is composed of 55 patients with primary squamous cell carcinoma of the head-and-neck (stage I-IVb) treated between 2006 and 2014 at Hôpital général juif, Montreal, QC. Included patients were treated with curative intent with radiation alone or with chemo-radiation. Patients with recurrent head-and-neck cancer or with metastases at presentation, and patients receiving palliative treatment were excluded from the study. The median follow-up period of the cohort was 46 months (range: 11-112). Patients that did not develop a locoregional recurrence or distant metastases during the follow-up period and that had a follow-up time smaller than 24 months were also excluded from the study. The study has been approved by the institutional review board of Hôpital général juif.

**Treatment Details**

Patients with stage I-II disease were treated with definitive radiotherapy alone while patients with stage III-IV disease were treated using concurrent chemo-radiation. The radiotherapy regimen was planned using Volumetric Arc Modulated Radiotherapy Rapidarc planning system (Varian Medical Systems). The radiotherapy regime consisted of hypofractionated fractionated radiotherapy with simultaneous integrated boost where the GTV was planned to receive a total of 67.5 Gy in fractions of 2.25 Gy over 6 weeks, while CTV received a total of 54-60 Gy in fractions of 1.8-2 Gy over 30 fractions. The treatment was delivered on a Linac equipped with HD120 Multileaf Collimator, with Image Guided Radiotherapy using daily kv-kv imaging and weekly Cone beam CT-scan (CBCT). Concomitant chemotherapy was given via weekly administration of Carboplatin at AUC 2-3 and Paclitaxel at dose of 40 mg/m^2^.

**^18^F-FDG PET/CT Scans**

All 55 eligible patients had FDG-PET and CT scans done on a hybrid PET/CT scanner (Discovery ST, GE Healthcare) within 37 days before treatment (median: 14 days). For the PET portion of the FDG-PET/CT scan, a median of 584 MBq (range: 368-715) was injected intravenously. Imaging acquisition of the head and neck was performed using multiple bed positions with a median of 300 s (range: 180-420) per bed position. Attenuation corrected images were reconstructed using an ordered subset expectation maximization (OSEM) iterative algorithm and a span (axial mash) of 5. The FDG-PET slice thickness resolution was 3.27 mm for all patients and the median in-plane resolution was 3.52×3.52 mm^2^ (range: 3.52-4.69). For the CT portion of the FDG-PET/CT scan, an energy of 140 kVp with an exposure of 12 mAs was used. The CT slice thickness resolution was 3.75 mm and the median in-plane resolution was 0.98×0.98 mm^2^ for all patients. Contours defining the gross tumour volume (GTV) and lymph nodes were drawn by an expert radiation oncologist in a radiotherapy treatment planning system. For 2 of the 55 patients, the radiotherapy contours were directly drawn on the CT scan of the FDG-PET/CT scan. For 53 of the 55 patients, the radiotherapy contours were drawn on a different CT scan dedicated to treatment planning. In the latter case, the contours were propagated to the FDG-PET/CT scan reference frame using deformable registration with the software MIM® (MIM software Inc., Cleveland, OH).

## Hôpital Maisonneuve Rosemont de Montréal

**Patient Population**

This cohort is composed of 26 patients with primary squamous cell carcinoma of the head-and-neck (stage II-IVb) treated between 2008 and 2014 at Hôpital Maisonneuve Rosemont, Montreal, QC. Included patients were treated with curative intent with radiation alone or with chemo-radiation. Patients with recurrent head-and-neck cancer or with metastases at presentation, and patients receiving palliative treatment were excluded from the study. The median follow-up period of the cohort was 38 months (range: 6-70). Patients that did not develop a locoregional recurrence or distant metastases during the follow-up period and that had a follow-up time smaller than 24 months were also excluded from the study. The study has been approved by the institutional review board of Hôpital Maisonneuve Rosemont.

**Treatment Details**

The treatment options consisted of either definitive radiotherapy alone or concurrent chemo-radiation. All patients received continuous course of radiotherapy delivered by a 6 MV linear accelerator using 7 to 9 elds inverse planning IMRT. Only one patient was planned with 5 elds and another was treated using 6 elds forward planning IMRT to the upper neck and direct anterior eld with a spinal cord block to the lower neck. For the patients receiving radiotherapy alone, 4 patients had stage II disease including a T1N1 nasopharyngeal cancer and received a dose 69.96 Gy in 33 fractions, 2 oropharyngeal and 1 hypopharyngeal cancer receiving altered fractionation with a dose of 66 to 67.5 Gy in 30 fractions. The 3 patients were offered but declined the chemotherapy and received 69.36 Gy in 33 fractions. Among patients receiving chemo-radiation, the radiation fractionation mostly used was 69.96 Gy in 33 fractions (n = 31) and the remaining received 70 Gy in 35 fractions (n = 2).

**^18^F-FDG PET/CT Scans**

All 26 eligible patients had FDG-PET and CT scans done on a hybrid PET/CT scanner (Discovery STE, GE Healthcare) within 60 days before treatment (median: 34 days). For the PET portion of the FDG-PET/CT scan, a median of 475 MBq (range: 227-859) was injected intravenously. Imaging acquisition of the head and neck was performed using multiple bed positions with a median of 360s (range:120-360) per bed position. Attenuation corrected images were reconstructed using an ordered subset expectation maximization (OSEM) iterative algorithm and a median span (axial mash) of 5 (range: 3-5). The FDG-PET slice thickness resolution was 3.27 mm for all patients and the median in-plane resolution was 3.52×3.52 mm^2^ (range:3.52-5.47). For the CT portion of the FDG-PET/CT scan, a median energy of 140 kVp (range: 120-140) with a median exposure of 11 mAs (range: 5-16) was used. The CT slice thickness resolution was 3.75 mm for all patients and the median in-plane resolution was 0.98×0.98 mm^2^ (range: 0.98-1.37). For all 26 patients, the radiotherapy contours defining the gross tumour volume (GTV) and lymph nodes were drawn by an expert radiation oncologist on a different CT scan dedicated to treatment planning. The contours were then propagated to the FDG-PET/CT scan reference frame using deformable registration with the software MIM® (MIM software Inc., Cleveland, OH).
